# The differences in virus shedding time between the Delta variant and original SARS-CoV-2 infected patients

**DOI:** 10.3389/fpubh.2023.1132643

**Published:** 2023-07-24

**Authors:** Fanglin Li, Jiayi Deng, Canbin Xie, Guyi Wang, Min Xu, Chenfang Wu, Jinxiu Li, Yanjun Zhong

**Affiliations:** ^1^Critical Care Medicine, The Second Xiangya Hospital, Central South University, Changsha, China; ^2^Department of Hematology and Critical Care Medicine, The Third Xiangya Hospital, Central South University, Changsha, China

**Keywords:** SARS-CoV-2, delta variant, COVID-19, virus shedding time, risk factor

## Abstract

**Background:**

The worldwide epidemic of Coronavirus Disease 2019 (COVID-19) has evolved into multiple variants. The Delta variant is known for its ability to spread and replicate, while data are limited about the virus shedding time in patients infected by the Delta variant.

**Methods:**

56 Delta variant and 56 original SARS-CoV-2 infected patients from Hunan, China, matched according to age and gender divided into two groups and compared the baseline characteristics and laboratory findings with appropriate statistical methods.

**Results:**

Patients infected with the Delta variant had significantly fewer symptoms of fever (*p* < 0.001), fatigue (*p* = 0.004), anorexia (*p* < 0.001), shortness of breath (*p* = 0.004), diarrhea (*p* = 0.006), positive pneumonia rate of chest CT (*p* = 0.019) and chest CT ground glass opacities (*p* = 0.004) than those of patients with the original SARS-CoV-2. Patients of the Delta variant group had a significantly longer virus shedding time [41.5 (31.5, 46.75) vs. 18.5 (13, 25.75), *p* < 0.001] compared with the original SARS-CoV-2 group. The correlation analyses between the virus shedding time and clinical or laboratory parameters showed that the virus shedding time was positively related to the viral strain, serum creatinine and creatine kinase isoenzyme, while negatively correlated with lymphocyte count, total bilirubin and low-density lipoprotein. Finally, the viral strain and lymphocyte count were thought of as the independent risk factors of the virus shedding time demonstrated by multiple linear regression.

**Conclusion:**

COVID-19 patients infected with the Delta variant exhibited fewer gastrointestinal symptoms and prolonged virus shedding time than those infected with the original SARS-CoV-2. Delta variant and fewer lymphocyte were correlated with prolonged virus shedding time.

## 1. Introduction

At the end of 2019, the world became aware of the outbreak of human infection with severe acute respiratory syndrome coronavirus 2 (SARS-CoV-2). The pandemic has continued till now and has not yet subsided. SARS-CoV-2 has developed diverse variations as a result of the viral genome’s ongoing evolution (1), which has brought immense challenges to the prevention and treatment of Coronavirus Disease 2019 (COVID-19).

The SARS-CoV-2 variants Alpha (B.1.1.7), Beta (B.1.351), Gamma (P.1), and Delta (B.1.617.2) have been classified by the World Health Organization (WHO) as variants of concern. Delta variant with considerable transmissibility and immune evasion capabilities have drawn a lot of attention (1, 2), were detected in India in October, 2020. It has been reported that the duration of viable viral shedding in confirmed patients may be influenced by new variants and SARS-CoV-2 vaccination strategies (3, 4). Patients with COVID-19 infected the Delta variant showed more prolonged viable viral shedding than those infected non-Delta variants before occurring the Delta variant (5). In addition, the duration of viral shedding is an important indicator for assessing the epidemic disease transmissibility (6, 7). Many studies have discussed risk factors associated with prolonged virus shedding time in COVID-19 patients (8–16). However, the relation between prolonged virus shedding time and SARS-CoV-2 variants remains unclear.

The outbreak in Hunan in August 2021 was caused by the Delta variant, while in 2020 was caused by the original SARS-CoV-2. This study aims to compare the characteristics of COVID-19 patients infected by the Delta variant and the original virus, especially discussing the independent risk factor of the difference between the two groups in the virus shedding time.

## 2. Methods and materials

### 2.1. Design and participants

The study is a retrospective observational study, which enrolled 56 Delta variant and 56 original COVID-19 patients matched with age and gender using propensity score matching, who were hospitalized in Hunan in 2020 to 2021. Each group includes 34 males and the average age is 41 years old.

### 2.2. Reverse transcription polymerase chain reaction

Throat swab samples were used to extract the nucleic acid by an automatic system from Nathch CS (Sansure Biotech, Hunan, China) and Mingde (Wuhan, China) for the Original and Delta variants, separately. Then, the nucleic acid amplification was conducted on slan96P (Shanghai Hongshi Medical Technology Co., LTD) and Yari MA-6000 (Mingde, Wuhan, China) separately.

### 2.3. Data collection

Epidemiological characteristics, comorbidities, clinical symptoms, radiography findings, medication history, baseline laboratory findings, and disease severity during hospitalization were extracted from the electronic medical records. The diagnosis of COVID-19 was based on RT-PCR results. Virus shedding time was defined as the duration between symptoms onset for symptomatic patients or first positive COVID-19 RT-PCR for asymptomatic cases and the first negative COVID-19 RNA (Ct ≥ 40) before discharge. Fever was defined as an axillary temperature ≥ 37.3°C. The definition of severe COVID-19 patients was determined according to the Chinese diagnosis and treatment protocol for novel coronavirus pneumonia (17). Data extraction was performed independently by two researchers, and a third researcher was brought in to assess if there were any discrepancies. This study was approved by The Institutional Ethics Board of The Second Xiangya Hospital of Central South University and Renmin hospital of Zhangjiajie, Hunan.

### 2.4. Statistical analyses

The quantitative variables were found to be non-normally distributed and were described as median (interquartile range, IQR). Comparisons between groups of independent quantitative variables were performed using Mann–Whitney test for the non-normal distribution. Categorical variables were described as frequency and percentage, and Fisher’s exact test or chi-square test were used for analyses. The Spearman correlation was applied to assess the correlations between virus shedding time and other variables. Multiple linear regression analysis was conducted to determine potential factors associated with virus shedding time included in the variables with *p* < 0.05 in the correlation analysis. All tests were two-sided and a *value of p* of <0.05 was considered statistically significant. Data analysis was performed using SPSS software (SPSS 26.0; IBM, United States) and the figure was designed using RStudio (Version 1.2.5001; RStudio, United States).

## 3. Results

The baseline characteristics of the enrolled 112 patients are shown in Table 1. The patients in the Delta variant and the original SARS-CoV-2 groups were matched according to gender and age. The Delta variant group exhibited a higher proportion of smoking (12.50% vs. 1.79%, *p* = 0.041), as well as lower incidences of fever (30.36% vs. 71.43%, *p* < 0.001), fatigue (17.86% vs. 44.64%, *p* = 0.004), anorexia (5.36% vs. 42.86%, *p* < 0.001), shortness of breath (7.41% vs. 30.36%, *p* = 0.004), and diarrhea (3.57% vs. 23.21%, *p* = 0.006). The Delta variant group also had significantly lower proportions of chest CT positive for pneumonia (75.00% vs. 92.86%, *p* = 0.019) and chest CT ground glass opacities (42.86% vs. 69.64%, *p* = 0.004) compared to the original SARS-CoV-2 group. What is particularly noteworthy is that the virus shedding time of the Delta variant group was dramatically longer than the original SARS-CoV-2 group [41.5 (31.5, 46.75) vs. 18.5 (13, 25.75) day, *p* < 0.001], despite a significantly higher vaccination rate in the Delta variant group compared to the Original group (80.36% vs. 0%, *p* < 0.001).

**Table 1:** Baseline characteristics of Delta variant and original SARS-CoV-2 infected patients.

	Delta variant (*n* = 56)	Original (*n* = 56)	*p* value
Gender [male, *n* (%)]	34 (60.71%)	34 (60.71%)	1.000
Age [year, median (IQR)]	41 (33.25, 56)	41 (35, 56.5)	0.766
Smoking / [*n* (%)]	7 (12.50%)	1 (1.79%)	**0.041**
Drinking / [*n* (%)]	1 (1.79%)	1 (1.79%)	1.000
Comorbidities			
Hypertension / [*n* (%)]	9 (16.07%)	9 (16.07%)	1.000
Coronary disease / [*n* (%)]	0 (0%)	0 (0%)	–
Diabetes / [*n* (%)]	6 (10.71%)	3 (5.36%)	0.469
Symptoms			
Fever / [*n* (%)]	17 (30.36%)	40 (71.43%)	**< 0.001**
Fatigue / [*n* (%)]	10 (17.86%)	25 (44.64%)	**0.004**
Cough / [*n* (%)]	41 (73.21%)	44 (78.57%)	0.659
Anorexia / [*n* (%)]	3 (5.36%)	24 (42.86%)	**< 0.001**
Chills / [*n* (%)]	6 (10.71%)	8 (14.29%)	0.776
Myalgia / [*n* (%)]	3 (5.36%)	6 (10.71%)	0.487
Shortness of breath / [*n* (%)]	4 (7.41%)	17 (30.36%)	**0.004**
Abdominal pain / [*n* (%)]	3 (5.36%)	3 (5.36%)	1.000
Sore throat / [*n* (%)]	12 (21.43%)	12 (21.43%)	1.000
Diarrhea / [*n* (%)]	2 (3.57%)	13 (23.21%)	**0.006**
Nausea / [*n* (%)]	0 (0%)	5 (8.93%)	0.067
Dizziness / [*n* (%)]	5 (8.93%)	6 (10.71%)	1.000
Headache / [*n* (%)]	6 (10.71%)	9 (16.07%)	0.580
Vomiting / [*n* (%)]	1 (1.79%)	5 (8.93%)	0.208
Chest CT with positive pneumonia / [*n* (%)]	42 (75.00%)	52 (92.86%)	**0.019**
Chest CT ground glass lesion / [*n* (%)]	24 (42.86%)	39 (69.64%)	**0.004**
Corticoid / [*n* (%)]	6 (10.71%)	15 (26.79%)	0.051
Anti-viral therapy / [*n* (%)]	30 (53.57%)	21 (37.50%)	0.088
Globulin/ [*n* (%)]	6 (10.71%)	15 (26.79%)	0.051
Severe disease / [*n* (%)]	3 (5.36%)	8 (14.29%)	0.204
Virus shedding time [day, median (IQR)]	41.5 (31.5, 46.75)	18.5 (13, 25.75)	**< 0.001**

IQR, interquartile range. Bold values means that *p* < 0.05 and is statistically significant.

The laboratory findings at initial admission of these two groups were contrasted (Table 2). Patients in the Delta variant group had lower lymphocyte counts (Lys) [0.95 (0.72, 1.36) vs. 1.30 (0.95, 1.73) x10^9^/L, *p* = 0.001], and lower erythrocyte sedimentation rates (ESR) [20.50 (12.25, 32.25) vs. 35.00 (18.50, 66.00) mm/h, *p* = 0.003] than those in the original SARS-CoV-2 group. In terms of liver function, the total bilirubin (TBil) [8.20 (6.50, 11.20) vs. 10.94 (7.35, 16.51) μmol/L, *p* = 0.009] of the Delta group was significantly lower while albumin (Alb) [44.60 (41.05, 45.40) vs. 38.95 (35.95, 42.10) g/L, *p* < 0.001] was significantly higher than the original SARS-CoV-2 group. With regard to the renal function, cardiac function, and blood lipid levels, the Delta variant group showed higher levels of creatinine (Cr) [70.00 (56.28, 82.65) vs. 46.69 (39.86, 57.00) μmol/L, *p* < 0.001], creatine kinase isoenzyme (CK-MB) [10.90 (8.60, 13.48) vs. 8.70 (5.00, 12.20) U/L, *p* = 0.015], and high-density lipoprotein (HDL) [1.12 (0.85, 1.30) vs. 0.81 (0.71, 0.98) mmol/L, *p* < 0.001], as well as a lower level of low-density lipoprotein (LDL) [2.31 (1.90, 2.52) vs. 2.62 (2.13, 3.20) mmol/L, *p* < 0.001], compared to the original SARS-CoV-2 group.

**Table 2:** Laboratory findings of Delta variant and original SARS-CoV-2 infected patients.

	Delta variant (*n* = 56)	Original (*n* = 56)	*p* value
WBC, x10^9^/L	4.93 (3.82, 5.99)	4.75 (3.97, 5.59)	0.718
Lys, x10^9^/L	0.95 (0.72, 1.36)	1.30 (0.95, 1.73)	**0.001**
ALT, U/L	23.00 (14.00, 34.00)	16.2 (13.17, 25.96)	**0.177**
AST, U/L	25.00 (20.00, 32.00)	23.34 (18.07, 32.58)	0.470
TBil, μmol/L	8.20 (6.50, 11.20)	10.94 (7.35, 16.51)	**0.009**
Alb, g/L	44.60 (41.05, 45.40)	38.95 (35.95, 42.10)	**< 0.001**
CRP, mg/L	9.56 (5.34, 16.97)	10.63 (3.00, 21.45)	0.731
ESR, mm/h	20.50 (12.25, 32.25)	35.00 (18.50, 66.00)	**0.003**
PCT, nmol/L	0.00 (0.00, 0.04)	0.00 (0.00, 0.05)	0.678
D-dimer, μg/mL	0.30 (0.25, 0.44)	0.23 (0.13, 0.52)	0.163
Cr, μmol/L	70.00 (56.28, 82.65)	46.69 (39.86, 57.00)	**< 0.001**
CK, U/L	68.10 (47.30, 92.43)	63.10 (46.40, 115.55)	0.990
CK-MB, U/L	10.90 (8.60, 13.48)	8.70 (5.00, 12.20)	**0.015**
TG, mmol/L	1.13 (0.76, 1.74)	1.08 (0.74, 1.54)	0.519
TC, mmol/L	3.68 (3.35, 4.19)	3.77 (3.24, 4.43)	0.882
LDL, mmol/L	2.31 (1.90, 2.52)	2.62 (2.13, 3.20)	**0.005**
HDL, mmol/L	1.12 (0.85, 1.30)	0.81 (0.71, 0.98)	**< 0.001**

WBC, white blood cell; Lys, lymphocyte; ALT, alanine aminotransferase; AST, aspartate aminotransferase; TBil, total bilirubin; CRP, C-reactive protein; PCT, procalcitonin; ESR, erythrocyte sedimentation rate; Alb, albumin; Cr, serum creatinine; CK, creatine kinase; CK-MB, creatine kinase isoenzyme; TG, triglyceride; TC, total cholesterol; LDL, low-density lipoprotein; HDL, high-density lipoprotein. Bold values means that *p* < 0.05 and is statistically significant.

**Figure 1:**
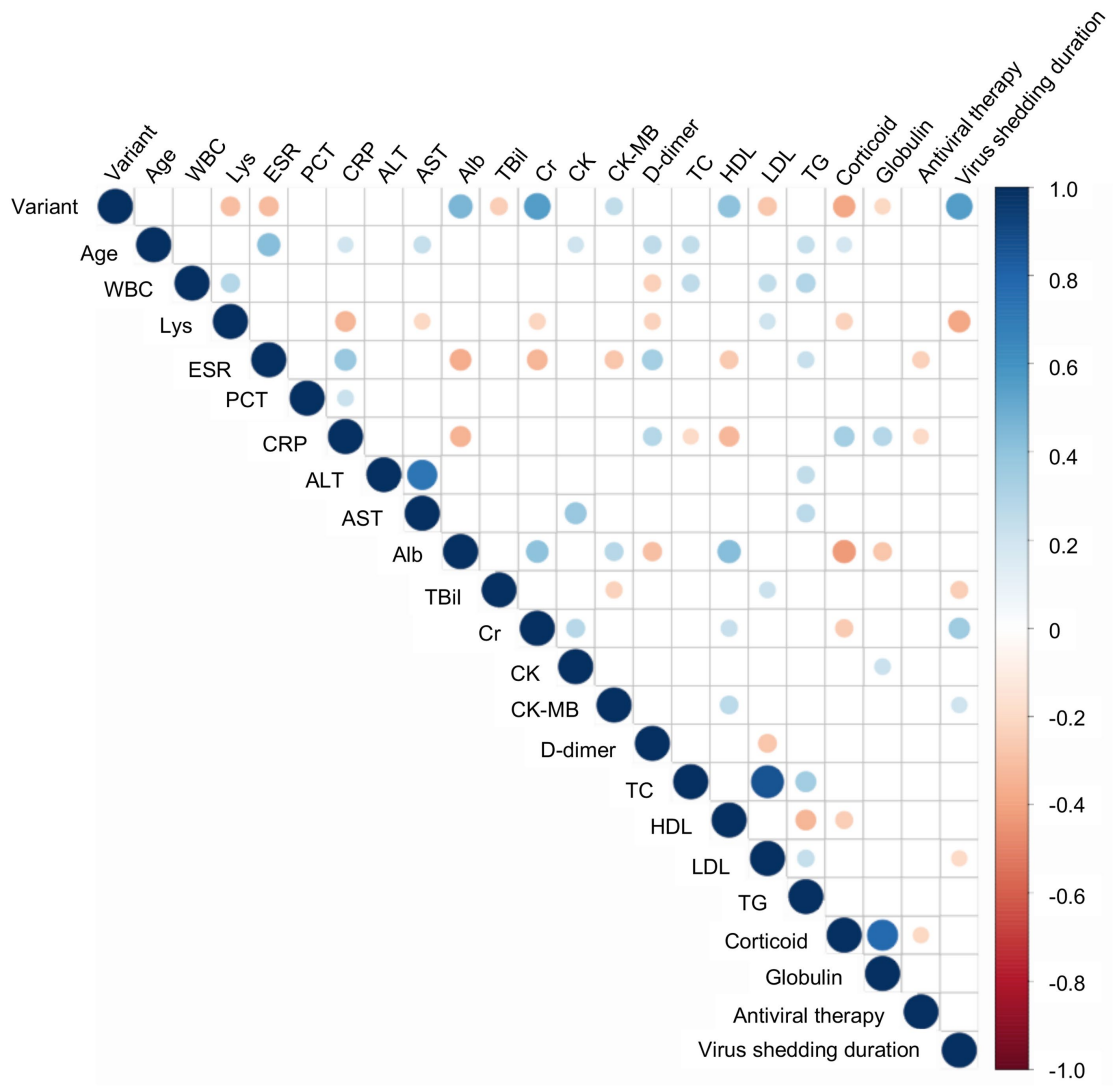
The correlation analyses between virus shedding time and other variables. Digits represent Spearman correlation coefficients, filled color indicates a significant correlation, blue represents a positive correlation, red represents a negative correlation, and the color’s shade represents the correlation’s strength. WBC, White blood cell count; Lys, Lymphocyte; ESR, Erythrocyte sedimentation rate; PCT, Procalcitonin; CRP, C-reactive protein; ALT, Alanine aminotransferase; AST, Aspartate aminotransferase; TBil, Total bilirubin; Cr, Creatinine; Chol, Cholesterol; HDL, High density lipoprotein; LDL, Low density lipoprotein; TG, Triacylglycerol.

Most important of all, we conducted correlation analyses to identify the potentially relevant factors that may impact virus shedding time. As illustrated in Figure 1, the virus strains, Cr and CK-MB were positively correlated with virus shedding time. The Delta variant was related to the prolonged virus shedding time. The virus shedding time was inversely correlated with Lys, TBil, and LDL. We further utilized multiple linear regression to determine the risk factors for virus shedding time (Table 3). The results indicated statistically significant associations between virus shedding time with the virus strains, as well as Lys count.

**Table 3:** Multiple linear regression model for virus shedding time of COVID-19 patients.

	*B*	SE	Beta	*T*	*p* value
COVID-19 strains	15.641 (9.820, 21.462)	2.930	0.524	5.338	**< 0.001**
Lys	−5.087 (−9.660, −0.514)	2.302	−0.196	−2.210	**0.030**
TBil	−0.178 (−0.663, 0.308)	0.244	−0.064	−0.728	0.468
Cr	0.004 (−0.107, 0.115)	0.056	0.007	0.072	0.942
CK-MB	0.001 (−0.379, 0.382)	0.192	0.001	0.007	0.994
LDL	0.766 (−2.650, 4.181)	1.719	0.040	0.445	0.657

Lys, Lymphocyte; TBil, Total bilirubin; Cr, Serum creatinine; CK-MB, Creatine kinase isoenzyme; LDL, Low density lipoprotein. Bold values means that *p* < 0.05 and is statistically significant.

## 4. Discussion

To the best of our knowledge, this is the first age– and gender-matched study to compare the differences between the COVID-19 caused by the Original strain and the Delta variant, which focuses on virus shedding time and related factors in patients with COVID-19. In the present study, COVID-19 patients infected with the Delta variant tended to have fewer gastrointestinal symptoms. More significantly, we found that the Delta variant may result in prolonged virus shedding, and that lower Lys counts were correlated with a longer virus shedding time.

Consistent with previous studies, the prevalence of smoking habits was significantly higher in patients infected with the Delta variants than in other variants (18). This suggests that smokers may be susceptible to the Delta variant. The study found that the most common symptoms of patients infected with the Delta variant were cough and fever, compared to the Original SARS-CoV-2, which was similar to other studies (19, 20). However, previous studies described that the clinical characteristics of patients infected with the Delta variant were not significantly different from the symptoms caused by other strains (1). In the present study, the patients infected with the Delta variant had fewer symptoms of fever, fatigue, shortness of breath, and digestive symptoms (anorexia and diarrhea). Furthermore, chest CT scans showed fewer cases of pneumonia and ground glass opacities, which could make it more indistinguishable from influenza and harder for patients to take seriously enough to seek timely medical attention. These finding align with a study in Spain, which reported that there was a higher prevalence of fever and gastrointestinal problems in patients infected with the original variant compared to those with the Delta variant. However, patients with the Delta variant showed higher neurological symptoms (21). A recent retrospective research of 107 patients revealed that the peak of pneumonia occurred later in the Delta variant than those with the Original COVID-19 (22), whereas our data on chest CT scans were collected upon admission. Notably, patients infected with the Delta variant had a significantly prolonged virus shedding time, which could lead to persistent medical deterioration. Replication-competent SARS-CoV-2 shedding was associated with immunocompromised state. Therapy strategies, e.g., corticoid and arbidol were also reported as independent risk factors for prolonged viral shedding time (9, 12). However, another study showed that lack of treatment with Paxlovid was an independent risk factor for prolonged viral shedding time (23). COVID-19 patients who started antiviral therapy later than 7 days experienced prolonged viral shedding time, compared to within 7 days after symptom onset (24). Nevertheless, our data did not reveal any significant differences in the uses of corticoid and anti-viral therapy between the Delta variant group and the original SARS-CoV-2 group, but the data collected for this study did not have a breakdown of the different types of antiviral drugs nor did it explain at what point viral therapy is initiated. Therefore, the present study did not further analyze their impact on viral shedding time.

Lymphocyte count is a sensitive indicator for evaluating the immune response of the body and a decreased count was correlated with disease severity in patients with COVID-19 (11). Our study showed that patients infected with the Delta variant had lower lymphocyte counts and ESR levels, suggesting that the Delta variant may impair immune function and induce a weaker non-specific inflammatory response, which may also explain the lower proportion of fever in these patients. In addition, we observed relatively higher levels of ALB and HDL, along with lower levels of TBil and LDL in patients infected with the Delta variant compared to those infected with the original SARS-CoV-2. Our previous research has shown that low HDL level and high TBil level may be correlated with a poor prognosis in patients with original COVID-19 (25, 26). Given the large-scale vaccination program that was already underway in China When the Delta variant emerged in 2021, the above changes of hematological parameters may be explained by the protective effect of vaccines (27). Finally, we observed that patients infected with the Delta variant tended to have higher levels of Cr and CK-MB, although these levels remained within the normal range.

Due to its important significance, we focused on exploring the risk factors associated with virus shedding time. It has been reported in 2020 that virus shedding time is an important factor in assessing transmission risk and is associated with fatal outcomes (6). In recent years, numerous studies have emerged discussing virus shedding time in COVID-19 patients since the outbreak of COVID-19. Some studies have found that older age (6, 11, 28) and male sex (10, 28) are risk factors for prolonged virus shedding time, while others have suggested that females are related to prolonged virus shedding time (8, 13). Prospective cohort studies have shown that vaccination reduces the risk of Delta variant infection and shortens virus shedding time (3, 29). We matched these two groups of patients according to age and gender to mitigate potential effects and use a multiple linear regression model to further clarify the factors that influence virus shedding time and determine their relationship. The result showed a linear correlation between viral strain and virus shedding time, with significantly prolonged virus shedding time in patients with the Delta variant. In addition, immune status also can influence the virus shedding (30, 31). For healthy individuals, Delta COVID-19 patients with higher levels of immunity recover from the virus more quickly and have a shorter virus shedding duration. Conversely, some studies suggest that immunocompromised individuals may shed infectious virus significantly longer not only in original but also Delta COVID-19 (31, 32).

It is known to us that the Delta variant exhibits a high level of transmission and pathogenicity, along with strong replication ability (33, 34). Furthermore, several studies have suggested that the viability of the delta variant SARS-CoV-2 is not solely dependent on Ct levels (8). Despite varying viral loads among infected individuals, severe cases of delta variant infection have been observed to result in prolonged virus shedding times. This could potentially be attributed to a greater number of mutations and an enhanced ability to evade the immune system (35). It is extremely valuable to characterize the virus shedding time for the development of effective quarantine guidelines and regulations to combat the Delta variant.

Certainly, there are also some limitations regarding the design and data in our study. First, it is a retrospective study and analyzed the data from a province at different times. Second, the sample size of the study is relatively small and it may restrict the ability clarify to and confirm differences between the Delta variant and other SARS-CoV-2 variants. Third, given the greater awareness of COVID-19 during the Delta wave, patients may have presented earlier and thus had a longer observation period resulting in an apparently longer duration of viral shedding. Forth, the use of viral shedding as a measure of viral viability in COVID-19 patients is not ideal. Viral shedding is usually measured by PCR detection of viral nucleic acid. However, viral nucleic acid testing does not entirely reflect the infectivity of the virus as it cannot differentiate between active and non-active virus particles (such as viral fragments) (36). The assessment of viral viability entails a holistic evaluation, which includes viral nucleic acid test results, the time window of infection, and the patients’ immune system (36–38). Lastly, the data collected for this study did not have a breakdown of the different types of anti-viral drugs/ time of anti-viral initiation. Therefore, further well-designed studies with larger sample sizes are needed to explore factors associated with the duration of viral shedding and deeper mechanics in patients with COVID-19.

## 5. Conclusion

COVID-19 patients infected with the Delta variant exhibited fewer gastrointestinal symptoms and prolonged virus shedding time than those infected with the original SARS-CoV-2. Delta variant and fewer lymphocyte were correlated with prolonged virus shedding time.

## Data availability statement

The original contributions presented in the study are included in the article/supplementary material, further inquiries can be directed to the corresponding authors.

## Ethics statement

The studies involving human participants were reviewed and approved by The Second Xiangya Hospital of Central South University and Renmin hospital of Zhangjiajie, Hunan. The patients/participants provided their written informed consent to participate in this study.

## Author contributions

YZ and JL developed the study design and conducted the analyses. FL and JD interpreted the results, reviewed the study design, and performed the statistical analyses. CX, GW, MX, and CW acquired patient demographic and clinical data. FL, YZ, JL did the revision of the manuscript. All authors contributed to the article and approved the submitted version.

## Conflict of interest

The authors declare that the research was conducted in the absence of any commercial or financial relationships that could be construed as a potential conflict of interest.

## Publisher’s note

All claims expressed in this article are solely those of the authors and do not necessarily represent those of their affiliated organizations, or those of the publisher, the editors and the reviewers. Any product that may be evaluated in this article, or claim that may be made by its manufacturer, is not guaranteed or endorsed by the publisher.
